# Successful treatment of pregnant women with syphilis and penicillin allergy

**DOI:** 10.1186/1939-4551-8-S1-A231

**Published:** 2015-04-08

**Authors:** Mara Morelo Rocha Felix, Sônia Hoana Silva, Cintia Bordalo, Marinauria Leal Pinto, Monica De Britto Pereira Bandeira De Mello, Jaqueline Ribeiro Toscano De Brito, Karla Do Carmo Ferrão, Jaqueline Coser Vianna, Raquel Grinapel, Andreia Albuquerque Garcês, Monica Soares De Souza, Aniela Bonorino Xexeo Castelo Branco

**Affiliations:** 1 Federal Hospital of Servidores Do Estado, Brazil

## Background

Congenital syphilis (CS) is a transplacentally transmitted infection caused by *Treponema pallidum* that occurs in infants of untreated or inadequately treated mothers. The preferred treatment for syphilis in pregnant mothers is penicillin. In patients with a history of penicillin allergy, skin testing and oral challenge should be performed. Penicillin desensitization is indicated for pregnant women with syphilis who demonstrate immediate hypersensitivity to this drug.

## Methods

We evaluated 6 pregnant women with syphilis and history of allergy to penicillin. They were submitted to ENDA (*European Network For Drug Allergy*) questionnaire and skin tests (ST), prick and intradermal, to benzylpenicillin 10.000 U/mL with histamine as positive control and saline as negative control. The reactions were considered positive when the size of the initial wheal increased by 3 mm or greater after 15 minutes. We performed oral provocation test (OPT) with penicillin V in case of negative penicillin ST. Patients with negative OPT received the first dose of benzathine penicillin G 2.400.000IU IM at the hospital under supervision.

## Results

Case 1: AV, 22 yo, VDRL 1:64. History of urticaria more than 1 hour after benzathine penicillin administration, nearly 9 months ago.

Case 2: ACSC, 16 yo, VDRL 1:32. History of angioedema after amoxicillin intake (unknown interval between intake and reaction). She had 2 episodes of allergic reaction (last one was 1 year ago).

Case 3: RSM, 38 yo, VDRL 1:64. History of urticaria minutes after benzathine penicillin administration at 14-years-old.

Case 4: GFL, 18 yo, VDRL 1:32. History of allergic reaction to penicillin during her first years of life (unknown interval between intake and reaction; unknown clinical manifestation).

Case 5: FAA, 16 yo, VDRL 1:1, positive TPHA. History of maculopapular exanthema 1 hour after benzathine penicillin administration. She had 2 episodes (at 5 and 12-years-old).

Case 6: MA, 29 yo, VDRL 1:4. History of allergic reaction at the site of the injection of benzathine penicillin 1h after administration. She had 2 episodes before 1 year-old.

All patients had negative tests (ST/OPT) and received treatment with penicillin without reactions.

## Conclusions

Adequate treatment of syphilis in pregnancy is crucial for prevention of CS. A reliable diagnosis is difficult in patients with a vague history of penicillin allergy. ST and OPT can be used for the evaluation of the hypersensitivity in order to decide whether desensitization is appropriate.

## Consent

Written informed consent was obtained from the patients for publication of this abstract and any accompanying images. A copy of the written consent is available for review by the Editor of this journal.

